# Dr. Edwin M. Kitchel

**Published:** 1897-09

**Authors:** 


Dr. Edwin M. Kitchel, assistant professor of histology and pathology in the College of Physicians and Surgeons, New York, died August 26, 1897, at Roosevelt Hospital from injuries received at the summer home of his father at Hulett's Landing, Lake George. Dr. Kitchel was playing a game of blindman's buff Tuesday evening, August 24th, on the piazza on the second floor of the house and while blindfolded fell over the railing, a distance of ten feet, sustaining an injury to his spine that resulted fatally. Dr. Frank M. Hupp, of Wheeling, West Virginia, was near by and gave what assistance was possible. It was decided to bring the injured man to New York and he was taken by boat to Caldwell and thence by train to New York. But nothing could be done to help him and he died an hour after his arrival at Roosevelt Hospital. Dr. Kitchel was the only son of James T. and Irene A. Kitchel, of Newark. He was graduated from Columbia University and the College of Physicians and Surgeons, and for four years had been the assistant of Dr. T. Mitchell Prudden, professor of histology and pathology in the latter institution. Dr. Kitchel was 26 years old. The funeral was held, August 28th, at the home of his father, at Riverside and Grafton avenues, Woodside, Newark.—*Tribune.*

